# A Replication-Defective Influenza Virus Vaccine Confers Complete Protection against H7N9 Viral Infection in Mice

**DOI:** 10.3390/vaccines8020207

**Published:** 2020-05-02

**Authors:** Shelby Landreth, Yao Lu, Kannupriya Pandey, Yan Zhou

**Affiliations:** 1 Vaccine and Infections Disease Organization, International Vaccine Centre (VIDO-InterVac), University of Saskatchewan, Saskatoon, SK S7N 5E3, Canada; shelby.landreth@usask.ca (S.L.); yao.lu@usask.ca (Y.L.); kannu.pandey@usask.ca (K.P.); 2 Vaccinology & Immunotherapeutics Program, School of Public Health, University of Saskatchewan, Saskatoon, SK S7N 2Z4, Canada; 3 Department of Veterinary Microbiology, Western College of Veterinary Medicine, University of Saskatchewan, Saskatoon, SK S7N 5B4, Canada

**Keywords:** influenza A virus H7N9, replication-defective virus vaccine, elastase dependent virus

## Abstract

Avian influenza H7N9 viruses continue to pose a great threat to public health, which is evident by their high case-fatality rates. Although H7N9 was first isolated in humans in China in 2013, to date, there is no commercial vaccine available against this particular strain. Our previous studies developed a replication-defective influenza virus through mutation of the hemagglutinin (HA) cleavage site from a trypsin-sensitive to an elastase-sensitive motif. In this study, we report the development of a reassortant mutant influenza virus derived from the human isolate A/British Columbia/01/2015 (H7N9) [BC15 (H7N9)], which is the QVT virus. The HA gene of this virus possesses three mutations at the cleavage site, Lys-Gly-Arg were mutated to Gln-Thr-Val at amino acid (aa) positions 337, 338, and 339, respectively. We report this virus to rely on elastase in vitro, possess unaltered replication abilities when elastase was provided compared to the wild type virus in vitro, and to be non-virulent and replication-defective in mice. In addition, we report this virus to induce significant levels of antibodies and IFN-γ and IL-5 secreting cells, and to protect mice against a lethal challenge of the BC15 (H7N9) virus. This protection is demonstrated through the lack of body weight loss, 100% survival rate, and the prevention of BC15 (H7N9) viral replication as well as the reduction of proinflammatory cytokines induced in the mouse lung associated with the influenza disease. Therefore, these results provide strong evidence for the use of this reassortant mutant H7N9 virus as a replication-defective virus vaccine candidate against H7N9 viruses.

## 1. Introduction

Avian influenza H7N9 viruses pose a significant threat to public health due to their recently acquired ability to infect humans and cause serious morbidity with high case-fatality rates (CFR). As of 4 March 2020, H7N9 had infected 1568 humans and resulted in 616 deaths FAO [1]. Prior to 2013, the low pathogenic avian influenza (LPAI) H7N9 virus circulated solely within the poultry population. Unfortunately, 2013 was the year when the H7N9 virus jumped species from birds into humans [2]. Since then, avian influenza H7N9 has resulted in a total of five epidemic waves from 2013 to 2017, with the fifth wave resulting in the emergence of the highly pathogenic avian influenza (HPAI) H7N9 virus [3,4]. More importantly, these epidemic waves resulted in CFR ranging from 33% to 45% for LPAI H7N9 and HPAI H7N9, respectively [3]. Although cases of H7N9 have been mainly constrained to China, there have been reports in other countries such as Canada [2,5,6]. The prevalence of the H7N9 virus in poultry and the expanded host range [7], along with the mutagenic properties influenza retains to escape the immune system through antigenic drift and antigenic shift, highlight the risk H7N9 poses to public health [8].

To prevent an H7N9 viral infection, various types of vaccines have been developed. Of the two main types of vaccines available for H7N9 infection, which include the inactivated influenza vaccine (IIV) and the live attenuated influenza vaccine (LAIV), the H7 IIV was deemed safe but less immunogenic in animals and humans [9,10]. Conversely, the LAIV provides protection from H7N9 replication and transmission in mammals [11]. Research has shown that there are advantages LAIVs have over IIVs, such as enhanced protection rates, longer-lasting immune responses, and the ability to mimic a natural infection by stimulating the mucosal immune system. In addition, LAIVs do not need to depend on the egg-based system since they can be propagated in a cell-culture based system and are easier to administer due to their needle-free properties [12,13]. However, albeit the surplus advantages LAIVs possess, one disadvantage toward their use is the possibility of virulence reversion, which limits their widespread use [14]. Replication-defective virus vaccines are composed of viruses that lack one or more vital components to either their replication, synthesis, or assembly of the virion. These viruses, therefore, cannot replicate in vivo, but can be propagated in vitro when provided the appropriate component [15]. These vaccines possess the same advantages both IIVs and LAIVs possess, which makes them strong influenza A virus (IAV) vaccine candidates.

One of the main determinants for whether IAV can establish infection and replicate within the host is the binding of the virus to the host cell through the interaction of hemagglutinin (HA) and sialic acid receptors. This interaction is followed by receptor-mediated endocytosis and fusion of the virus to the host endosome. The HA of IAV has three important roles: it enables IAV to bind to the host cell, it induces fusion between the viral envelope and endosomal membranes allowing the release of viral ribonucleoproteins into the cytoplasm, and it is the primary antigen the immune system produces neutralizing antibodies against [16]. The HA of IAV is initially synthesized in its precursor form, HA0, which must be cleaved into HA1 and HA2 by a host protease. This cleavage is necessary for IAV to become infectious, and can play a role in the pathogenicity and tissue tropism [16,17,18]. We previously demonstrated that when the HA cleavage site was mutated from a trypsin-sensitive to an elastase-sensitive motif, influenza virus became replication-defective in vivo [16]. In this study, we report the development of a reassortant mutant H7N9 virus, QTV, which possesses the six internal genes from A/Puerto Rico/8/1934 (H1N1) [PR8 (H1N1)], and the wild type (WT) neuraminidase (NA) and mutated HA genes derived from the human isolate A/British Columbia/01/2015 (H7N9) [BC15 (H7N9)] through reverse genetics. The cleavage site of the HA gene was mutated from Lys-Gly-Arg to Gln-Thr-Val at amino acid (aa) positions 337, 338, and 339, respectively. This reassortant mutant virus relies on elastase for its replication in vitro, but is replication-defective in mice. Moreover, this reassortant mutant H7N9 virus was capable of inducing a strong immune response and completely protecting mice from a lethal challenge of BC15 (H7N9). Therefore, this reassortant mutant QTV virus holds strong promise as a replication-defective virus vaccine candidate against H7N9 influenza viral infection.

## 2. Materials and Methods

### 2.1. Cells and Viruses

Madin-Darby canine kidney (MDCK) (ATCC, #CRL-2936) cells and human embryonic kidney (HEK-293T) cells were cultured in Minimal Essential Medium (MEM) (Sigma-Aldrich, M4655, St. Louis, MO, USA) and Dulbecco’s Modified Eagle Medium (DMEM) (Sigma-Aldrich, D5796, St. Louis, Mo, USA), respectively, and supplemented with 10% fetal bovine serum (FBS) (Thermo Fisher, 16000-044) in a humidified 5% CO_2_ incubator at 37 °C. The A/British Columbia/01/2015 (H7N9) [BC15 (H7N9)] strain isolate was obtained from Dr. Yan Li at the National Microbiology Laboratory, Public Health Agency of Canada. This strain isolate was maintained in Biosafety Containment Level 3 at the Vaccine International Disease Organization, International Vaccine Centre (VIDO-InterVac,) at the University of Saskatchewan, Saskatoon, Canada, under the guidelines of the Public Health Agency of Canada (PHAC) and the Canadian Food Inspection Agency (CFIA). The reassortant wild type (rWT) H7N9 virus, which contains six internal genes from PR8 (H1N1) and the WT NA and HA genes from BC15 (H7N9), was grown in Madin-Darby Canine Kindney (MDCK) cells in the presence of 0.2% bovine serum albumin (BSA) (Sigma-Aldrich, A7030, St. Louis, MO, USA) and 1 μg/mL L-[(toluene-4-sulphonamido)-2-phenyl] ethyl chloromethyl ketone (TPCK)-trypsin. The reassortant mutant QTV virus, which contains six internal genes from PR8 (H1N1), and the WT NA and the mutated HA genes from BC15 (H7N9), was grown in MDCK cells supplemented with 0.2% BSA and 0.5 μg/mL human neutrophil elastase (Sigma-Aldrich, E8140, St. Louis, MO, USA).

### 2.2. Plasmids and the Generation of Reassortant Viruses

The WT HA and NA genes derived from BC15 (H7N9) virus were cloned into pHW2000 as previously described, which resulted in the plasmids pHW-BC15-HA and pHW-BC15-NA, respectively [19].

For the development of the mutant HA, pHW-BC15-HA underwent site-directed mutagenesis using the primers 5′ – ACA GAC TGT TGG ACT ATT TGG TGC TAT AGC GGG TTT C – 3′ and 5′ – TCC AAC AGT CTG TGG AAT CTC AGG AAC ATT CTT CAT C – 3′. The resulting plasmid, pHW-BC15-HA/QTV, encodes HA composed of three mutations at the HA cleavage site. Specifically, Lys-Gly-Arg were mutated to Gln-Thr-Val at amino acid (aa) positions 337, 338, and 339, respectively. The sequence of the plasmid was confirmed by DNA sequencing to ensure only the desired mutations were introduced by PCR. The plasmids generated were used to produce two reassortant viruses, rWT and QTV, through the use of the eight-plasmid reverse genetics system, as previously described [19]. The rWT virus contains six internal genes from PR8 (H1N1) and the WT NA and HA genes from BC15 (H7N9), whereas the QTV virus contains six internal genes from PR8 (H1N1), the WT NA and the mutated HA genes from BC15 (H7N9).

### 2.3. Western Blot Analysis

Cell lysates were separated by sodium dodecyl sulfate-polyacrylamide gel electrophoresis (SDS-PAGE) and transferred to nitrocellulose membranes (Bio-Rad, 0.45 μm, Hercules, CA, USA). These membranes were blocked for 1 h with 5% skim milk dissolved in TBST (0.1 M Tris, 0.17 M NaCl, and 1% Tween 20), washed twice with TBST, and incubated overnight at 4 °C with either rabbit anti-NP or rabbit anti-M1 antibodies raised in our lab. The next day, the membranes were washed four times with TBST, and then incubated with donkey anti-rabbit IgG (IR Dye 680RD, Li-Cor, Lincoln, NE, USA) at room temperature for 1 h, which was followed by the membranes being washed four times with TBST. Lastly, the membranes were scanned on an Odyssey infrared imager (Li-Cor Biosciences).

### 2.4. Plaque Assay and Viral Replication Curve

To determine the viral titres, MDCK cells (5 × 10^5^ cells) were plated on each well of a 6-well plate in MEM and 10% FBS. Twenty-four h later, the cells were infected with the virus at dilutions ranging from 10^−1^ to 10^−8^. After 1 h of viral adsorption, the cells were washed with MEM, and an agar overlay was added containing 0.9% agarose, MEM, 0.2% BSA, and either 1 μg/mL TPCK-trypsin (rWT virus) or 0.5 μg/mL human neutrophil elastase (reassortant mutant QTV virus). The cells were then incubated at 37 °C, 5% CO_2_ for 48 h. At 48 h, the agar overlay was removed and cells were stained with Coomassie Blue Stain. The plaques were counted and the viral titre was determined as a plaque-forming unit (PFU) per mL.

To determine the replication curve, MDCK cells were infected with the virus at an m.o.i. of 0.001. Aliquots were harvested at 12, 24, 36, 48, 60, and 72 h.p.i. and subjected to a plaque assay to determine the viral titre.

### 2.5. Ethics Statement

Before proceeding with the animal procedures, approval was required from the University Animal Care Committee (UACC) and the Animal Research Ethics Board (AREB) of the University of Saskatchewan. This approval was granted on 12 April 2019 (Animal Use Protocol #20190041) for the virulence trial of the reassortant mutant QTV virus, and on 18 June 2019 (Animal Use Protocol #20190079) for the immunogenicity and protective efficacy of the reassortant mutant QTV virus. The approvals granted were guided by the stipulations implemented from the Canadian Council on Animal Care (CCAC).

### 2.6. Mouse Trials and Sampling

For this study, two mouse trials were performed to assess: (1) the virulence of the reassortant mutant QTV virus, and (2) the immunogenicity as well as protective efficacy of the reassortant mutant QTV virus against a homologous challenge of BC15 (H7N9) (WT). For the first trial, 36 six-week-old male and female BALB/c mice (18 males and 18 females) (Charles River Laboratories, Saint-Constant, QC, Canada) were randomly divided into three groups with 12 mice in each group (six males and six females). These groups were housed in separate cages and allowed to acclimatize for seven days in Biosafety Containment Level 2. At seven weeks of age, the mice were intranasally inoculated with 50 μL of either MEM or the following reassortant viruses at 1 × 10^3^ PFU: rWT or QTV. The mice were monitored daily for body weight and survival rate. On day 3 post-infection (d.p.i.), four mice from each group (two males and two females) were humanely euthanized and their lungs were collected for viral titration. The rest of the mice were humanely euthanized when they dropped below 20% of their initial body weight.

For the second trial, 38 six-week-old male and female BALB/c mice (19 males and 19 females) (Charles River Laboratories, Saint-Constant, QC, Canada) were randomly divided into three groups with either 12 or 14 mice in each group (six or seven males and females, respectively). These groups were housed in separate cages and allowed to acclimatize for seven days in Biosafety Containment Level 2. The males and females were housed in separate cages within the groups. At seven weeks of age, the mice were intranasally vaccinated with 50 μL of either MEM or the QTV virus at a dose of 1 × 10^3^ PFU. At 10 weeks of age (21 days later), the mice received a second vaccination in the same manner as the first vaccination. On day 30, four mice from each group were euthanized (two males and two females) and their spleens collected for splenocyte isolation to detect IFN-γ and IL-5 secreting cells. The remaining eight or ten mice per group (four or five males and females, respectively) were transferred to Biosafety Containment Level 3. The following day (day 31), these transferred mice were challenged with a lethal dose 100 (LD100) of BC15 (H7N9) (WT) (1 × 10^3^ PFU). Afterward, the mice were monitored daily for 14 days for both body weight and survival rate. On day 3 post-challenge, four mice per group (two males and two females) were humanely euthanized and their lungs and serum were collected. Any mouse that lost over 20% of its total initial body weight throughout the trial was humanely euthanized. In addition, the serum of all mice was collected on days 0, 21, and 30. All animal experiments took place at VIDO-InterVac, University of Saskatchewan following the regulations stipulated by the University of Saskatchewan and the CCAC.

### 2.7. Detection of IFN-γ and IL-5 Secreting Cells by Enzyme-Linked Immunospot (ELISPOT) Assay

Multiscreen-HA plates, 0.45 μM, sterile (Millipore, MAHAS4510) were coated with either purified rat anti-mouse IFN-γ (BD, 551216) or purified rat anti-mouse IL-5 (BD, 554393) overnight at 4 °C. Mouse splenocytes were seeded at 5 × 10^5^ cells/well and were stimulated for 20 h at 37 °C with 50 μg/mL of either the purified β-propiolactone-inactivated rWT virus or medium only in triplicate. The spots were developed as described previously, and counted with the ELISPOT reader (AID, Strassberg, Germany) [20]. The data reported is presented as the number of IFN-γ and IL-5-secreting cells per 5 × 10^5^ cells with the number of spots in the medium-only wells subtracted as background.

### 2.8. Enzyme-Linked Immunosorbent Assay (ELISA) to Detect Antigen-Specific IgG, IgG1, and IgG2

Purified β-propiolactone-inactivated rWT virus (2.5 μg/mL) was coated onto 96-well Immulon-2 plates (Dynex Technology Inc., Chantilly, VA, USA) and incubated overnight at 4°C. Serially diluted mouse sera was added and left to incubate at room temperature for 1.5 h. Goat anti-mouse IgG (H+L) (Invitrogen, B2763, Carlsbad, CA, USA), goat anti-mouse IgG1 (Southern Biotech, 1070-08, Birmingham, AL, USA), or goat anti-mouse IgG2a (Southern Biotech, 1080-08, Birmingham, AL, USA) was added for 1 h at room temperature to detect IgG, IgG1, or IgG2a, respectively. Color development was initiated by the addition of alkaline phosphatase (AP)-conjugated streptavidin (Jackson ImmunoResearch) and p-nitrophenyl phosphate (PNPP) substrate [10 mg/mL p-nitrophenyl phosphate di (tris) salt crystalline (Sigma-Aldrich, St. Louis, MO, USA), 1% diethanolamine (Sigma-Aldrich, St. Louis, MO, USA), 0.5 mg/mL MgCl_2_, pH 9.8]. Detection of the optical density (OD) occurred at 405 nm (using a reference filter of 490 nm) on a microplate reader (Molecular Devices SpectraMax Plus 384, San Jose, CA, USA). The titres of each sample were determined as the highest dilution at which the OD of the sample was larger than the defined cut-off. The cut-off was defined as the mean OD of a known negative sample plus twice the standard deviation.

### 2.9. Hemagglutination Inhibition (HAI) and the Serum Viral Neutralization (SVN) Assay

The hemagglutination inhibition (HAI) and serum viral neutralization (SVN) assays were conducted according to a previous report [20]. Four HA units of BC15 (H7N9) were used in the HAI assay.

### 2.10. Virus Isolation and Titration

Upon harvest of the mouse lung, the tissues were submerged in MEM supplemented with Penicillin-Streptomycin (Gibco, Thermo Fisher, ON, Canada). The tissues were homogenized in the TissueLyser II (Qiagen, Hilden, Germany) at 25 Hz for 5 min, which was followed by centrifugation at 5000 g for 10 min at 4 °C. The homogenized supernatant was transferred to new tubes and stored at −80 °C until analysis. The viral load was titrated by the fifty-percent tissue culture infective dose (TCID_50_) assay as described previously, and the titres were calculated using the Spearman-Kärber algorithm [21,22].

### 2.11. RNA Extraction and Quantitative RT-PCR (qRT-PCR)

The lung tissue harvested was immediately placed into RNAlater (Qiagen, Toronto, ON, Canada) and stored overnight at 4 °C. RNA was extracted using TRIzol (Invitrogen, Carlsbad, CA, USA), according to the manufacturer’s instruction.

For qRT-PCR, 1 μg of total RNA was reverse transcribed into cDNA with oligo(dT) and the superscript III reverse transcriptase (Invitrogen, 18080-044, Carlsbad, CA, USA). qPCR was performed with gene specific primers in the StepOnePlus^TM^ Real-Time PCR system (Applied Biosystems, Foster City, CA, USA) with the Power SYBR Green PCR Master Mix (Applied Biosystems, Foster City, CA, USA). The hypoxanthine phosphoribosyltransferase (HPRT) housekeeping gene was used to standardize the cytokine mRNA levels. All results are reported according to the ∆∆CT method using the mock challenge group as a reference [23]. All qPCR primer sequences are available upon request.

### 2.12. Statistical Analysis

The statistical analysis was executed using GraphPad Prism 8. For this analysis, a one-way ANOVA was performed, which was followed by the Tukey post-hoc test to determine the *p*-value. For the ELISPOT, ELISA, and qPCR, the samples were analyzed in triplicate. For the SVN assay, the samples were performed in quadruplicate. For the HAI assay, the samples were tested in duplicate. Significant differences among groups are signified by * (*p* < 0.05), ** (*p* < 0.01), *** (*p* < 0.001) or **** (*p* < 0.0001). ns. = not significant.

## 3. Results

### 3.1. The Generation of Two Reassortant H7N9 Viruses

**Figure 1:**
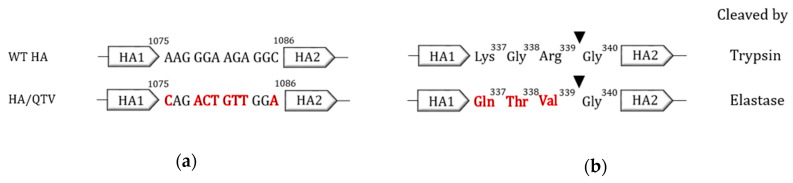
The schematic outline of the mutations introduced into the HA cleavage site of BC15 (H7N9). (**a**) Nucleotide sequences from HA positions 1075 to 1086 and (**b**) amino acid sequences from HA positions 337 to 340 of WT HA (wild type) and HA/QTV (mutant plasmids). Nucleotide sequences (**a**) and amino acids (**b**) in red correspond to the mutations that were introduced. Elastase corresponds to the human neutrophil elastase protease.

Two reassortant H7N9 viruses were generated. First, we generated the reassortant wild type (rWT) virus, which contains six internal genes from PR8 (H1N1) and the WT HA and NA genes from BC15 (H7N9). This virus could be rescued in the presence of trypsin. Second, we generated the reassortant mutant QTV virus, which comprises the same gene segments as that of the rWT virus, except the HA gene possesses three mutations at the cleavage site. Specifically, the nucleotides in the WT HA gene corresponding to positions 1075 to 1086 were mutated from AAG GGA AGA GGC to CAG ACT GTT GGA (Figure 1a). This exchange resulted in the replacement of the amino acids (aa) Lys-Gln-Arg at positions 337–339 with Gln-Thr-Val (Figure 1b) to result in the mutant plasmid HA/QTV. This virus was rescued in the presence of human neutrophil elastase.

### 3.2. The Reassortant Mutant QTV Virus Is Dependent on Elastase and Possesses Equivalent Replication Abilities as the WT Counterpart

**Figure 2:**
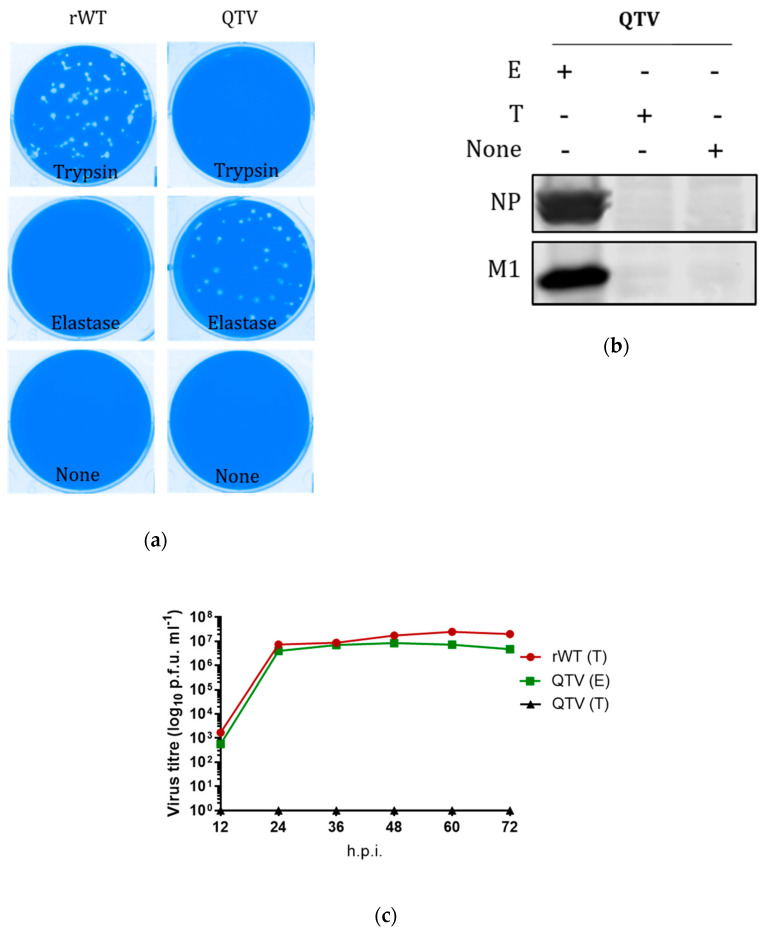
The generation and characterization of the reassortant mutant QTV virus in relation to its replication-dependency and kinetics. (**a**,**b**) The replication-dependency of reassortant wild type (rWT) and reassortant mutant QTV viruses. MDCK cells were infected at an m.o.i. of 0.001 in the presence of 1 μg/mL TPCK-trypsin, 0.5 μg/mL human neutrophil elastase, or in the absence of an exogenous protease. The supernatant and cells were harvested at 48 h.p.i., and underwent either plaque assay (**a**) or Western Blotting (**b**) to detect the presence of nucleoprotein (NP) and matrix (M1) proteins. (**c**) The replication curve of the rWT and QTV viruses on MDCK cells. The cells were infected with the respective virus at an m.o.i. of 0.001 with either 1 μg/mL TPCK-trypsin (T) or 0.5 μg/mL human neutrophil elastase (E). The reassortant mutant QTV virus was also tested in the presence of TPCK-trypsin. The supernatants were collected at specified time points until 72 h.p.i., and then titered by plaque assay on MDCK cells.

To determine the elastase dependency of the reassortant mutant QTV virus in vitro, we performed the plaque assay and Western blotting. The reassortant mutant QTV virus as well as the rWT virus were assayed in the presence of trypsin, human neutrophil elastase, or no protease. The reassortant mutant QTV virus formed similar-sized plaques in the presence of elastase as did the rWT virus. Either virus could not form plaques in the absence of a protease (Figure 2a). Viral NP and M1 proteins were detectable in the QTV-infected MDCK cells only when elastase was supplemented (Figure 2b).

Since high replication abilities are one of the requirements for a virus to be considered a good vaccine candidate for the purpose of vaccine manufacturing, we wanted to determine whether the introduction of the mutations in the HA cleavage site could result in altering the replication abilities of the reassortant mutant QTV virus. To this end, a multicycle replication curve was conducted in MDCK cells (Figure 2c). The reassortant mutant QTV virus exhibited similar replication kinetics to its rWT counterpart with rapid replication between 12 h and 24 h, which plateaued after 24 h. When trypsin was added to the reassortant mutant QTV virus growth medium in the absence of elastase, no viral replication was detected.

### 3.3. The Reassortant Mutant QTV Virus Is Non-Virulent and Replication-Defective in Mice

**Figure 3:**
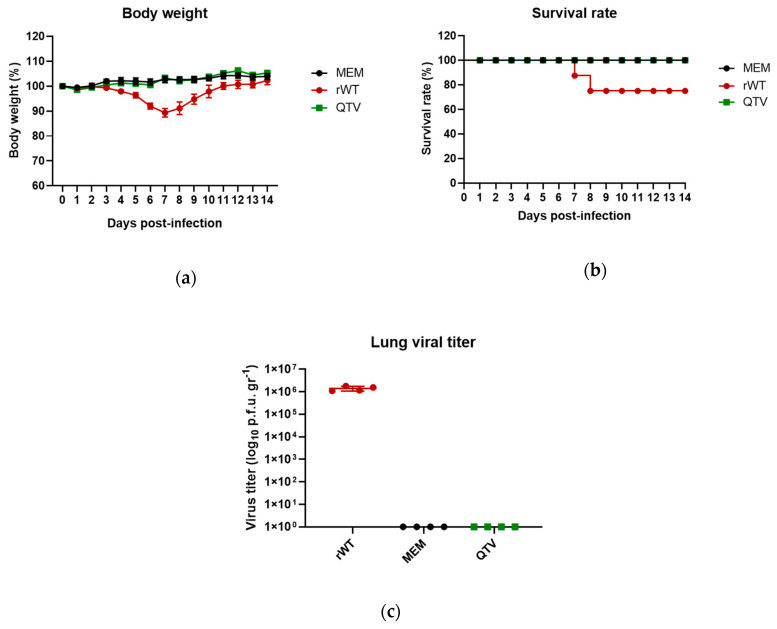
The body weight, survival rate, and lung viral titration of mice infected with the rWT virus and the reassortant mutant QTV virus. Male and female BALB/c mice (*n* = 12 per group, equal males and females) were intranasally infected with either MEM (control), the rWT virus, or the reassortant mutant QTV virus at a single dose of 1 × 10^3^ PFU. The mice were monitored daily, with any mouse reaching a humane intervention point to be humanely euthanized and their lung samples harvested. (**a**) The body weights. (**b**) The survival rate. (**c**) The viral titration of homogenized lung samples at 3 d.p.i. by plaque assay. The samples were analyzed in duplicate. Viral titres are expressed as PFU per gram (PFU/gr). The data is shown as the mean ± the standard deviation. The dots correspond to the values obtained from individual mouse samples.

To assess the replication potential and virulence of this reassortant mutant QTV virus, 36 six-week-old male and female BALB/c mice (equal males and females) were randomly divided into three groups with 12 mice in each group (six males and six females). Each mouse was intranasally inoculated with 50 μL of either MEM (mock-infected), rWT virus (1 × 10^3^ PFU), or the reassortant mutant QTV virus (1 × 10^3^ PFU). The mice were monitored daily for body weight and survival rate (Figure 3a,b). On 3 d.p.i., four mice from each group (two males and two females) were humanely euthanized and the lung samples were taken for viral titration (Figure 3c). The mice that were mock-infected with MEM as well as the mice that were infected with the reassortant mutant QTV virus survived the duration of the trial and did not show any body weight loss. In contrast, the mice that were infected with the rWT virus lost weight from 4 d.p.i., with the most weight loss occurring on 7 d.p.i. Two mice (one female and one male) succumbed to infection on 7 and 8 d.p.i., respectively. In agreement with this observation, on 3 d.p.i., high viral replication was detected in the rWT infected group (mean titre of 1 × 10^6^ PFU/gr), whereas no viral replication was detected in the reassortant mutant QTV infected group.

### 3.4. The Reassortant Mutant QTV Virus Induces Robust Antibody Responses

**Figure 4:**
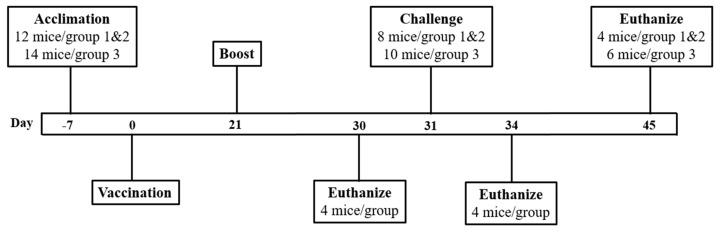
The outline of mice vaccinated with the reassortant mutant QTV virus and challenged with the homologous BC15 (H7N9) virus. BALB/c mice (*n* = 12 or 14, equal males and females) were intranasally vaccinated on days 0 and 21 with 50 μL of MEM (control) or the QTV virus (1 × 10^3^ PFU). On day 30, four mice per group (two males and two females) were euthanized for splenocyte isolation. On day 31, the remaining mice were intranasally challenged with MEM (control) or a lethal dose of BC15 (H7N9) (WT) (1 × 10^3^ PFU). On day 34 (three days post-challenge), four mice per group (two males and two females) were euthanized for sampling. The trial concluded on day 45 (14 days post-challenge), where the remaining mice were euthanized and sampled. Sampling included serum and lung tissue.

**Figure 5:**
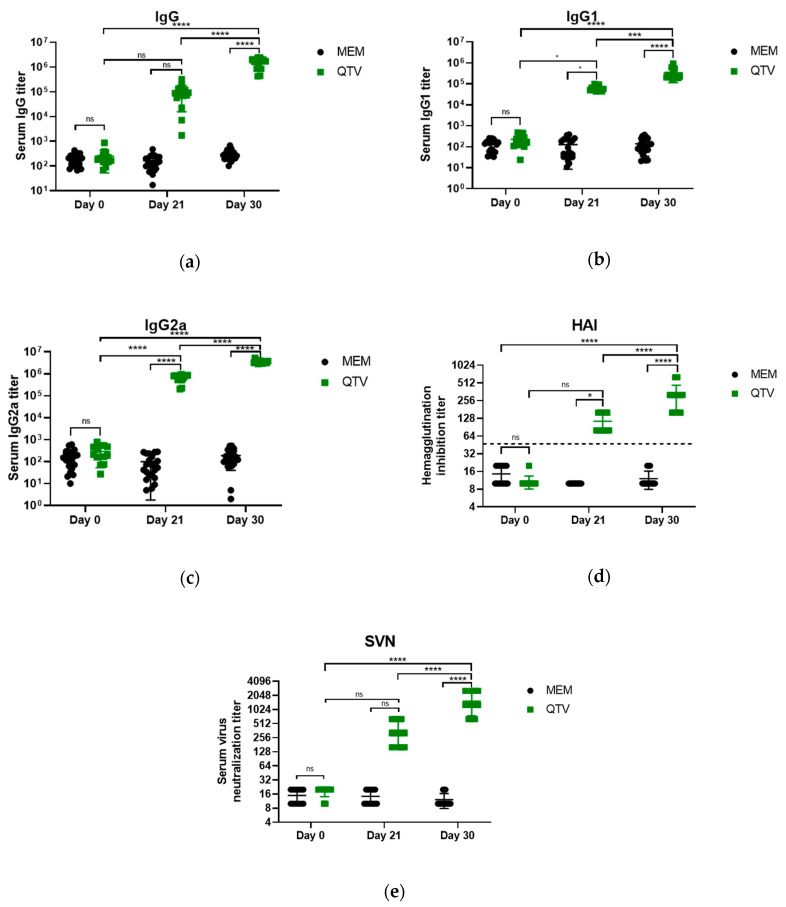
Antibody responses mounted after QTV vaccination. BALB/c mice were vaccinated on days 0 and 21 with MEM (control) or the QTV virus at a dose of 1 × 10^3^ PFU. Serum was collected on days 0, 21, and 30 to be titered using an ELISA to detect influenza-specific IgG, IgG1, and IgG2a. Samples were analyzed in triplicate. Serum was also titered for neutralizing antibodies by the HAI assay and SVN assay. (**a**) IgG. (**b**) IgG1. (**c**) IgG2a. (**d**) Serum HAI titres were determined using 0.5% red blood cells (RBCs) against the BC15 (H7N9) (WT) virus. The samples were analyzed in duplicate. The dotted line corresponds to the negative cut-off of 40, which is associated with a 50% reduction in the chance of contracting the influenza virus. (**e**) SVN titres were determined using 100 TCID_50_/50 μL of the BC15 (H7N9) influenza virus. The samples were analyzed in quadruplicate. The data is shown as the mean ± the standard deviation. The dots correspond to the values obtained from individual mouse samples. Significant differences among groups are signified by * (*p* < 0.05), *** (*p* < 0.001) or **** (*p* < 0.0001). ns. = not significant. A one-way ANOVA was performed, which was followed by the Tukey post-hoc test to determine the *p*-value.

To determine the immune response and protection efficacy of the replication-defective QTV virus, male and female BALB/c mice were intranasally immunized on days 0 and 21 with MEM (mock-vaccinated) or 1 × 10^3^ PFU of the QTV virus (vaccinated). On day 31, these mice were challenged with a lethal dose (LD100) of BC15 (H7N9) (WT) virus (1 × 10^3^ PFU/mouse) (Figure 4) [24]. Mouse serum was collected before vaccination (day 0), three weeks after the first vaccination (day 21), and nine days after the second vaccination (day 30). Influenza-specific IgG, IgG1, and IgG2a in the mouse serum were determined for days 0, 21, and 30 by ELISA using purified β-propiolactone-inactivated rWT virus as the capture antigen. As seen in Figure 5a, the levels of IgG were dramatically elevated after the first QTV vaccination when compared to the day 0 levels (day 0 titre = 249, day 21 titre = 98,101). After the second vaccination, IgG levels were significantly upregulated when compared to the levels after the first vaccination (day 30 titre = 1,502,010) (*p* < 0.0001). Similarly, the levels of IgG1 after the first QTV vaccination were significantly upregulated (*p* = 0.0464) in comparison to the day 0 levels (day 0 titre = 225, day 21 titre = 65,428) (Figure 5b). After the second QTV vaccination, the levels of IgG1 increased significantly (titre for day 30 = 335,175) in comparison to day 21 (*p* < 0.0001) and day 0 (*p* < 0.0001). In contrast, the levels of IgG2a after the first QTV vaccination were significantly upregulated (*p* < 0.0001) when compared to the day 0 levels (day 0 titre = 290, day 21 titre = 677,648) (Figure 5c). After the second vaccination, the levels of IgG2a were significantly upregulated in comparison to day 21 levels (day 30 titre = 3,601,440) (*p* < 0.0001). Therefore, although significant upregulation was observed for influenza-specific IgG, IgG1, and IgG2a after two vaccinations, the dramatic upregulation observed after a single vaccination in mice may be sufficient to confer protection against a lethal challenge of the BC15 (H7N9) virus.

Concerning the HAI assay, all mice were negative on day 0 for BC15 (H7N9) antibodies (HAI ≤ 1:16), and the mice that were mock-vaccinated with MEM remained negative on days 21 and 30 for BC15 (H7N9) antibodies (HAI ≤ 1:16) (Figure 5d). After the first vaccination (day 21) with QTV, all the mice seroconverted to the BC15 (H7N9) antigen with an average HAI level of 128. This level of seroconversion was substantially higher than the gold standard HAI titre cut-off of 40 that is associated with a 50% reduction in the risk of contracting influenza [25]. Following the second vaccination (day 30), the level of seroconversion in the QTV-vaccinated mice increased significantly and reached an average HAI titre of 256. Consistently, the SVN assay demonstrated that after the first vaccination (day 21), SVN titres increased dramatically and reached an average SVN titre of 256. Moreover, after the second vaccination (day 30), the SVN titres increased significantly to reach an average SVN titre of 1024 when compared to the day 21 levels (*p* < 0.0001). Therefore, these results are in agreement with the influenza-specific IgG, IgG1, and IgG2a results in that, although significant upregulation was observed after two vaccinations, the dramatic upregulation after a single vaccination may be sufficient to confer protection against the BC15 (H7N9) virus.

### 3.5. The Reassortant Mutant QTV Virus Produces High Numbers of Both IFN-γ and IL-5 Antigen-Specific Secreting Cells

**Figure 6:**
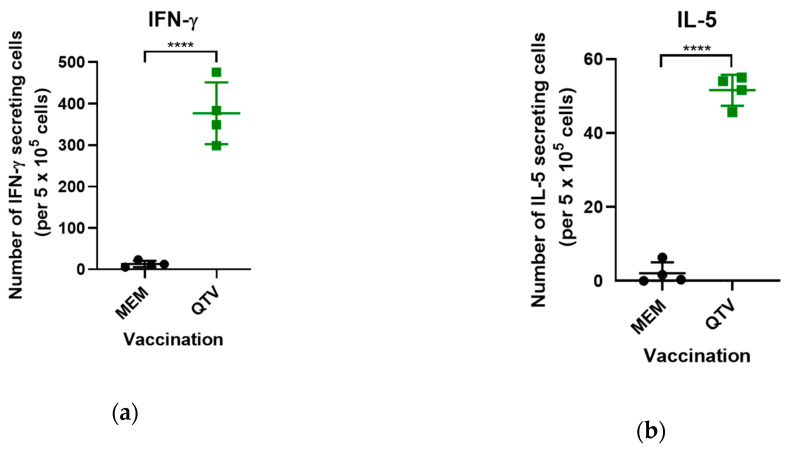
Antigen-specific IFN-γ and IL-5 secreting cells induced by QTV vaccination in mice. BALB/c mice were vaccinated with two doses of MEM (control) or QTV virus (1 × 10^3^ PFU). Nine days after the second vaccination (day 30), BALB/c mice (four mice per group, two males and two females) were euthanized and their splenocytes were isolated. The numbers of IFN-γ (**a**) and IL-5 (**b**) secreting T cells per 5 × 10^5^ splenocytes were determined by ELISPOT. Each sample was tested in triplicate. The antigen-induced counts were determined by subtracting the number of IFN-γ or IL-5 secreting cells with the medium-treated control cells. The data is shown as the mean ± the standard deviation. The dots correspond to the values obtained from individual mouse samples. Significant differences among groups are signified by **** (*p* < 0.0001). ns = not significant. A one-way ANOVA was performed, which was followed by the Tukey post-hoc test to determine the P-value.

The mouse spleens were harvested on day 30 from both the MEM mock-vaccinated and QTV vaccinated mice (two males and two females per group). The splenocytes were isolated and the antigen-specific responses were measured by both the IFN-γ and IL-5 ELISPOT assay. Intranasal vaccination with QTV induced significantly higher numbers of antigen-specific IFN-γ secreting cells compared to the mock-vaccinated MEM control group (*p* < 0.0001) (Figure 6a). Concomitantly, the number of antigen-specific IL-5 secreting cells induced by QTV vaccination, although not as high as IFN-γ, was still significantly higher when compared to the mock-vaccinated MEM control group (*p* < 0.0001) (Figure 6b).

### 3.6. Vaccination with the Reassortant Mutant QTV Virus Completely Protects Mice from a Lethal Challenge of BC15 (H7N9)

**Figure 7:**
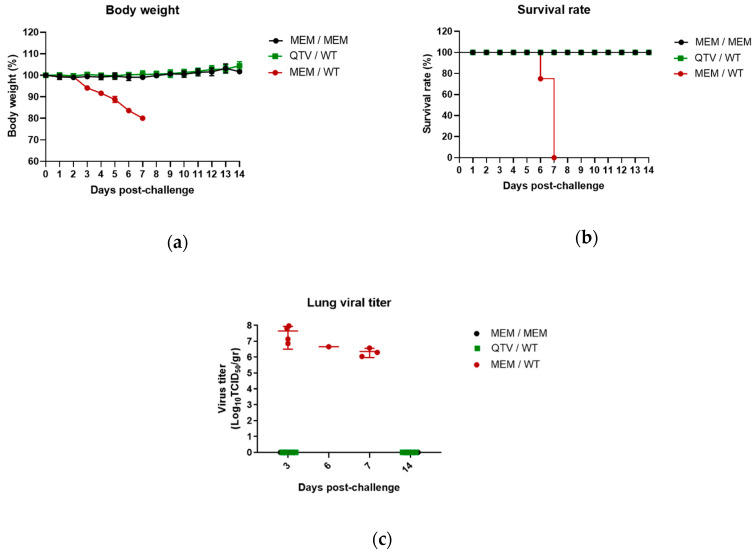
The body weight, survival rate, and lung viral titration of vaccinated mice after viral challenge with the BC15 (H7N9) virus. BALB/c mice were intranasally vaccinated with two doses of MEM (control) or QTV virus (1 × 10^3^ PFU). These mice were then intranasally challenged with BC15 (H7N9) (WT) at a lethal dose of 1 × 10^3^ PFU. [*n* = eight per group, four males and four females for MEM / MEM and MEM / WT], [*n* = 10 per group, five males and five females for QTV / WT]. (**a**) The body weight. (**b**) The survival rate. (**c**) The viral titration of homogenized lung samples. Viral titration of the homogenized lung tissue was conducted by the TCID_50_ assay. The samples were analyzed in quadruplicate. Four mice per group were humanely euthanized three days post-challenge, while the remaining mice were euthanized 14 days post-challenge. Dots correspond to the individual mouse lung titres. The four dots on three days post-challenge correspond to the four mice that were humanely euthanized. The one dot on six days post-challenge corresponds to one mouse that succumbed to infection. The three dots on seven days post-challenge correspond to three mice that succumbed to infection. The data is shown as the mean ± the standard deviation. The dots correspond to the values obtained from individual mouse samples.

After the BC15 (H7N9) (WT) viral challenge, the mice were monitored for 14 days for body weight loss and survival rate (Figure 7a,b). Mice that were mock-vaccinated and mock-challenged with MEM survived the duration of the trial, slightly gaining weight as the days progressed. Likewise, the mice that were vaccinated with QTV and challenged with BC15 (H7N9) survived the duration of the trial, and gained weight as the days progressed. Conversely, the mice that were mock-vaccinated with MEM and challenged with BC15 (H7N9) demonstrated rapid weight loss, reaching a humane endpoint of weight loss greater than 20% of their initial body weight within 6-days and 7-days post-challenge.

In agreement with these results, infectious virus could not be detected from the lungs of QTV vaccinated and BC15 (H7N9) challenged mice 3 days and 14 days post-challenge. In contrast, the mice mock-vaccinated with MEM and challenged with BC15 (H7N9) displayed high lung mean viral loads three days post-challenge (10^7.65^ TCID_50_/gr), six days post-challenge (10^6.65^ TCID_50_/gr), and seven days post-challenge (10^6.36^ TCID_50_/gr), with all the mice succumbing to infection by seven days post-challenge (Figure 7c).

### 3.7. Vaccination with the Reassortant Mutant QTV Virus Reduces the Production of Proinflammatory Cytokines in the Mouse Lung Associated with the Influenza H7N9 Viral Infection

**Figure 8:**
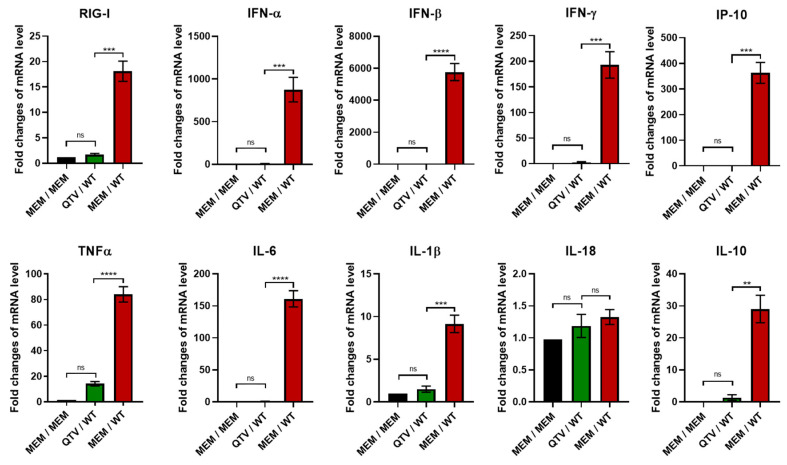
Cytokine production after homologous viral challenge with BC15 (H7N9). The mRNA levels of RIG-I, IFN-α, IFN-β, IFN-γ, IP-10, TNFα, IL-6, IL-1β, IL-18, and IL-10 were determined from the mouse lungs harvested at three days post-challenge (*n* = four mice per group, two males and two females). These levels were assessed by qPCR, where each sample was tested in triplicate. The bars represent the mean value obtained from all mice in the respective group, and the error bar represents the standard deviation. Significant differences among groups are signified by ** (*p* < 0.01), *** (*p* < 0.001) or **** (*p* < 0.0001). ns. = not significant. A one-way ANOVA was performed, which was followed by the Tukey post-hoc test to determine the *p*-value.

We previously showed that BC15 (H7N9) viral infection induces substantial gene upregulation of the innate immune receptor retinoic acid-inducible gene I (RIG-I), interferons (IFN), and proinflammatory cytokines in mice, which contributes to viral pathogenicity [24]. To confirm that vaccination with the reassortant mutant QTV virus prevented mice from pathological immune responses caused by viral infection, we evaluated the gene expressions of RIG-I, IFNs, and proinflammatory cytokines by qRT-PCR (Figure 8).

While the mRNA levels of RIG-I were significantly upregulated in the mice mock-vaccinated with MEM and challenged with BC15 (H7N9) (WT) (over 15-fold), mRNA levels in the mice vaccinated with QTV and challenged with BC15 (H7N9) were comparable to the mice mock-vaccinated and mock-challenged with MEM. Type I IFNs (IFN-α and IFN-β), type II IFNs (IFN-γ), and interferon-gamma-inducing protein 10 (IP-10) were all significantly upregulated in the mice mock-vaccinated with MEM and challenged with BC15 (H7N9) (IFN-α around 1000-fold, IFN-β around 6000-fold, IFN-γ around 200-fold, and IP-10 over 300-fold). Conversely, the levels of IFN-α, IFN-β, IFN-γ, and IP-10 remained at much lower levels in the mice vaccinated with QTV and challenged with BC15 (H7N9) when compared to the mice mock-vaccinated and challenged with BC15 (H7N9). Similarly, the mRNA levels of proinflammatory cytokines TNFα, IL-6, and IL-1β, as well as anti-inflammatory cytokine IL-10, displayed similar trends, in that significant upregulation was observed in the mice mock-vaccinated with MEM and challenged with BC15 (H7N9) (TNFα around 80-fold, IL-6 around 150-fold, IL-1β around 10-fold, and IL-10 around 30-fold). There was no significant change in the mice vaccinated with QTV and challenged with BC15 (H7N9) when compared to the mock-vaccinated and mock-challenged group. There was no significant change for IL-18 for all groups tested.

## 4. Discussion

Replication-defective influenza virus vaccines possess the combined advantages of IIVs and LAIVs, with regards to their high safety and efficacy rates, low possibility of reversion, and ability to stimulate a balanced immune response [14,15]. Some strategies to create a replication-defective influenza virus include alternation of the genetic codon [26], deletion of the IFN antagonist NS1 gene [27], knock-out of the PB2 gene [28], and modification of the HA cleavage site. All these strategies are applied in either lab-adapted influenza virus or swine influenza virus. However, in this case, we report the generation of a replication-defective influenza virus vaccine candidate against the zoonotic H7N9 influenza virus, which poses a public health threat.

Our results showed that vaccination with the replication-defective QTV virus induces strong antibody responses, especially neutralizing antibodies. Additionally, immunized mice produce high numbers of both IFN-γ and IL-5 secreting splenocytes in response to influenza virus stimulation. It has long been believed that both the cell-mediated immunity and humoral immunity play essential, yet distinct roles in the control of influenza viruses. Humoral immunity has been known to entirely block and prevent influenza infection by producing antibodies against the influenza antigen [29,30]. More specifically, it has been shown that the most important antibody for the neutralization of influenza is the HA-specific antibody. This antibody functions by binding to the trimeric globular head of HA on the influenza virus to block attachment and entry of the virus into the host cell. Antibodies are also generated against the HA stem of influenza virus to a lesser extent [30]. On the contrary, cell-mediated immunity does not prevent viral entry but, instead, promotes viral clearance by activating T cells, specifically CD4^+^ and CD8^+^ T cells. CD8^+^ T cells are known to differentiate into cytotoxic T lymphocytes (CTLs) with their primary function being to aid in host defence by killing virus-infected cells. The production of IFN-γ has been shown to aid in the differentiation of CD8^+^ T cells into CTLs. Conversely, CD4^+^ T cells possess a more complex role, which differentiate into Th1 or Th2 cells upon influenza infection, to activate B cells, stimulate antibody production, and express antiviral cytokines such as IFN-γ. CD4^+^ T cells are also known to produce cytokines such as IL-4 and IL-5, known to promote antibody responses [31]. The production of IFN-γ, therefore, is essential to control the influenza virus within the host cell. IFN-γ is also known to possess antiviral activity and is one of the first cytokines produced in response to pathogen invasion, which leads to the activation of macrophages and NK cells to aid in controlling infection [32]. Moreover, the induction of cell-mediated immunity has been correlated with protection against the highly HPAI viruses, such as the H5N1 influenza virus. Several studies have demonstrated that the protection against the HPAI viruses can be entirely mediated by cellular immunity, even in the complete absence of humoral immunity [29]. In our study, vaccination with QTV was adequate to induce significant levels of IFN-γ secreting-cells in the mouse spleen (Figure 6). Simultaneously, vaccination also induced significant levels of IL-5 secreting-cells, however, to a lower extent when compared to IFN-γ. These results, therefore, indicate that this reassortant mutant QTV virus is capable of inducing T cell activation.

Analysis by the HAI assay demonstrated the production of neutralization antibodies above the gold standard HAI titre cut-off of 40 after both the first (HAI titre of 128) and second immunizations (HAI titre of 256) (Figure 5). Analysis by the SVN assay also demonstrated the high production of neutralization antibodies that could correlate with protection after the first (SVN titre of 256) and second immunizations (SVN titre of 1024). In agreement with the previous results, the levels of antigen-specific IgG, IgG1, and IgG2a were highly elevated after both the first and second immunizations. IgG2a and IgG1 are known to be immunoglobulin indicators for Th1 and Th2 responses, respectively [33]. A Th1 response is known to produce cytokines such as IFN-γ and IL-2, which aids in the production of the cell-mediated response. Conversely, a Th2 response aids in the production of IL-4, IL-5, and IL-10 and antibody production. Since IgG1 and IgG2a were both elevated after the first and second immunizations, this could potentially point to our replication-defective viral vaccine capable of inducing both Th1 and Th2 responses. This dual stimulation ability is advantageous in comparison to IIVs, which mainly stimulate humoral immunity [13].

Influenza viruses, such as H7N9, are known to be potent inducers of proinflammatory cytokines, which can lead to inflammation and damage of the lung epithelial cells [24]. One of the host’s first line of defence against influenza virus is inflammation, which if not regulated properly, can lead to substantial pathology and occasional death of the host [34]. In our study, BC15 (H7N9) induces significantly elevated levels proinflammatory cytokines and chemokines in the lungs of mice mock-vaccinated and challenged with BC15 (H7N9). However, this significant upregulation was avoided in the mice vaccinated with QTV and challenged with BC15 (H7N9) (Figure 8). This avoidance is consistent with the lung viral titres and the protection rates, which suggests that significant cytokine induction is correlated with the severity of BC15 (H7N9) infection. It is noted that IFN-γ production in the mice vaccinated with QTV and challenged with BC15 (H7N9) is 2.5-fold higher than that in the mock-vaccinated and mock-challenged mice. We rationalized that this level of IFN-γ is produced by the local lymphocytes in QTV vaccinated mice after being re-stimulated by the BC15 (H7N9) (WT) virus. This level of IFN-γ reflects the results shown in Figure 6, in that QTV vaccination induced the production of high numbers of IFN-γ secreting cells in the spleen, and benefits the host in preventing viral infection.

This vaccine’s innocuity is based on the QTV virus dependency on elastase to replicate. Elastase, which is a serine protease, is biologically capable of digesting elastin in order to maintain tissue elasticity [35]. In mammals, elastase is known to primarily reside within the pancreas and pancreatic juice [36,37]. Moreover, studies have shown that, in the human serum, the two primary sources of elastase are the lung neutrophils and alveolar macrophages [37,38]. Moreover, studies have demonstrated the usefulness of elastase toward the development of various influenza virus vaccines, discovering that these vaccines can be readily propagated in vitro when provided elastase, but are unable to replicate in vivo because there is no elastase readily available in the respiratory tract [39]. These studies have been demonstrated in mice and pigs [39,40,41,42,43,44,45]. This restricted replication also reduces the risk of virulence reversion, which demonstrates the high safety profile this QTV virus possesses over traditional LAIVs, whose limited replication poses a higher risk for virulence reversion [14,15]. Further research of this QTV virus should be conducted in other animal models, such as the ferret model, to establish the safety, immunogenicity, and protective efficacy. Further research should also be conducted to evaluate whether one vaccination is sufficient to induce protection as well as whether this QTV virus is protective against viral infection with other H7N9 strains, given its ability to stimulate the cell-mediated immunity.

## 5. Conclusions

In this study, we report the generation of an elastase-dependent replication-defective influenza virus as a potential vaccine candidate for H7N9 virus. We evaluated the vaccine innocuity, immunogenicity, and efficacy of the vaccine in protecting mice against a lethal challenge of BC15 (H7N9). Our results provide strong evidence for the use of this reassortant mutant H7N9 virus as a replication-defective virus vaccine candidate against H7N9 virus.
